# A Case of an Unusual Presentation of Hemoptysis: Oropharyngeal Leech

**DOI:** 10.7759/cureus.87587

**Published:** 2025-07-09

**Authors:** Maham R Sidhu, Muhammad A Hassan, Mubashir Rafique, Faryal Ghani

**Affiliations:** 1 Cardiology, Glenfield Hospital, Leicester, GBR; 2 General Surgery, Rawalpindi Medical University, Rawalpindi, PAK

**Keywords:** direct laryngoscopy, foreign body, leech, oropharynx, pharyngeal wall

## Abstract

We report a case of a 36-year-old male who presented with a complaint of blood-stained sputum for 15 days. The patient gave a history of drinking from a contaminated pool. On examination, there were blood streaks on the posterior pharyngeal wall. The patient’s vital and baseline blood investigations were within normal limits. On direct laryngoscopy under general anesthesia, a greenish-black foreign body was found moving in the oropharynx and was taken out by heated Magill forceps. It was identified as a leech. Through this case, we emphasize the need for well-set diagnostic criteria that can not only enhance the reporting of such cases but can also be used as a surveillance tool by the public health authorities.

## Introduction

Leech, a freshwater-inhabiting worm, is typically found in tropical areas. Its saliva contains anesthetic, anti-coagulant, and vasodilator properties, all of which make these organisms excellent blood-sucking creatures. People in rural areas present rarely after ingesting leeches through contaminated water [1].

## Case presentation

A 36-year-old male, a shepherd and farmer, was referred from a rural healthcare facility to an ENT tertiary care setup with a complaint of blood-stained sputum for 15 days. There was no associated history of fever, weight loss, night sweats, dysphagia or odynophagia, hematemesis, melena, or respiratory tract infection. The patient gave a history of taking contaminated water from a pond. On examination, blood streaks on the posterior pharyngeal along with small and multiple mucosal ulcerations were seen. He was vitally stable throughout his admission to the ward. All the baseline investigations were within normal limits, including normal hemoglobin.

**Figure 1:**
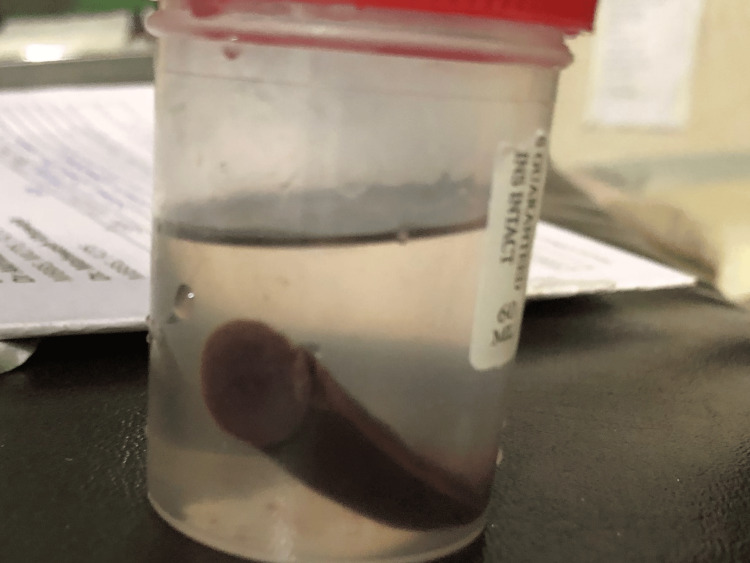
Isolated leech. The figure shows the leech removed from the patient. It was fairly big in size, approximately 7-8 cm in length.

On suspicion of a foreign body in the aerodigestive tract, the patient was shifted to the operating room for direct laryngoscopy under general anesthesia. The endotracheal tube was not passed. Upon direct laryngoscopy, a greenish-black foreign body in the oropharynx was seen, and it was removed by warm Magill forceps. It was identified as a leech approximately the size of 7-8 cm (Figure 1). The blood-stained sputum was not seen later on, and the patient was discharged the next day after an uneventful recovery.

## Discussion

Leeches are carnivorous, hermaphrodite, segmented worms that belong to the phylum Annelida, class Hirudinea. Aquatic leeches can live in both fresh and saltwater bodies, most commonly in relatively still waters, sluggish streams, and paddy fields, but there are also those that live on land. Leeches can vary from 5 to 15 cm in length. These leeches might enter the human body through external openings while drinking or bathing in the contaminated water and can lead to internal hirudiniasis [2].

The most common site for leech infestation is the nose, and the most common symptom encountered is epistaxis. It can rarely involve the aerodigestive tract. The pharyngeal leech manifestation can present with hemoptysis, a sense of a foreign body in the throat, dysphagia, and melena. Laryngeal involvement can subsequently lead to airway obstruction, which is an acute emergency, while in the long term, leech infestation can manifest as anemia [3].

The diagnosis of a disease without a proper history is very challenging, with diagnostic criteria or specific investigations yet to be developed. With the diagnostic dilemma, taking the leach out of the body is a difficult task for a surgeon who is not very experienced, owing to the rarity of the cases being reported. Some reports suggest removing it after injecting paralyzing agents like cocaine or lignocaine, while in our case, the practice of taking it out through a heated instrument was used, and it was successful.

As seen in our patient, the painless loss of blood during coughing in sputum in the form of streaks is an extremely rare manifestation of the disease. The patient’s normal vitals and hemoglobin and longer duration of the infestation are indicative of the fact that the pharyngeal manifestation is more of a local disease with no aggressive systemic complications.

## Conclusions

In a country like Pakistan, with a large population not having access to clean water, the rare number of cases being reported of this disease reflects the under-diagnosis rather than the decreased prevalence. The diagnostic criteria along with set guidelines for the management of leach infestation can result in an increased number of cases being reported. A good reporting system can alarm the local public health authorities for taking necessary precautions to help prevent the extent of the disease.
